# Expression of TLR-2, TLR-4, NOD2 and pNF-κB in a Neonatal Rat Model of Necrotizing Enterocolitis

**DOI:** 10.1371/journal.pone.0001102

**Published:** 2007-10-31

**Authors:** Aurelie Le Mandat Schultz, Arnaud Bonnard, Frédérick Barreau, Yves Aigrain, Coralie Pierre-Louis, Dominique Berrebi, Michel Peuchmaur

**Affiliations:** 1 Assistance Publique-Hôpitaux de Paris (AP-HP) Hôpital R. Debré, Service de Chirurgie Pédiatrique, Paris, France; 2 Université Paris7, EA3102, Paris, France; 3 Ecole de Chirurgie, Assistance Publique-Hôpitaux de Paris (AP-HP), Paris, France; 4 Assistance Publique-Hôpitaux de Paris (AP-HP) Hôpital Robert Debré, Service d'Anatomie et de Cytologie Pathologiques, Paris, France; 5 INSERM, U843, Hôpital Robert Debré, Paris, France; 6 UMR-S843, Université Paris Diderot, Paris, France; Oregon Health & Science University, United States of America

## Abstract

**Background:**

The etiology of necrotizing enterocolitis (NEC) results from a combination of several risk factors that act synergistically and occurs in the same circumstances as those which lead to innate immunity activation. Pattern recognition molecules could be an important player in the initiation of an exaggerated inflammatory response leading to intestinal injury in NEC.

**Methodology/Principal Findings:**

We specifically evaluated intestinal epithelial cell (IEC) expression of Toll-like receptor 2 (TLR-2), TLR-4, NOD2 and phosphorylated NF-κB (pNF-κB) after mucosal injury in a rat model of NEC induced by prematurity, systemic hypoxia, and a rich protein formula. In the control group (group 1), neonatal rats were full-term and breast-fed; in the experimental groups, rat pups were preterm at day 21 of gestation and rat-milk fed (group 2) or hand-gavaged with a protein rich formula after a hypoxia–reoxygenation procedure (group 3). Morphological mucosal changes in the small bowel were scored on hematoxylin- and eosin-stained sections. Immunohistochemistry was performed on frozen tissue sections using anti TLR-2 and active pNF-κB p65 antibodies. Real-time RT-PCR was performed to assess mRNA expression of NOD2, TLR-2 and TLR-4. Proliferation and apoptosis were studied in paraffin sections using anti Ki-67 and caspase-3 antibodies, respectively. The combination of immaturity, protein rich formula and a hypoxia–reoxygenation procedure induces pathological mucosal damage consistent with NEC. There was an overexpression of TLR-2, and pNF-κB in IECs that was correlated with the severity of mucosal damage, together with an increase of apoptotic IECs and markedly impaired proliferation. In addition, these immunological alterations appeared before severe mucosal damage. TLR-2 mRNA were also increased in NEC together with TLR-4 mRNA using real-time RT-PCR whereas NOD2 expression was unchanged.

**Conclusions/Significance:**

These results show that this rat model of NEC induced mucosal injury, leading to a highly responsive IEC phenotype and suggesting that alterations in the innate immune system participates in the pathogenesis of NEC and are enhanced by prematurity.

## Introduction

Necrotizing enterocolitis (NEC) is the main cause of mortality in multisystem organ failure in intensive care units and occurs predominantly among preterm and very low birth weight newborns [1], [2]. NEC mainly affect the distal ileum and proximal colon. The pathological features of NEC are transmural hemorrhagic necrosis and inflammatory infiltrates in advanced stages of disease and destruction of the mucosa in initial stages.

The outcome is the release of inflammatory mediators in the intestinal wall, which leads to variable degrees of intestinal damage. Increased proinflammatory cytokines are found in intestinal samples from NEC cases, suggesting that these mediators play a role in the development of NEC [3]–[8].

The pathogenesis of NEC is ill defined, but involves prematurity, hypoxia, the intake of cow's milk, and an inappropriate or immature mucosal response to the intestinal microflora. Indeed, NEC seems to occur within the first weeks after birth in a context of increased mucosal permeability due to intestinal barrier dysfunction [9]. Insofar as intestinal epithelial cells represent the first barrier against exogenous pathogens in the intestine, immature local innate defenses have also been suggested as a factor in the physiopathology of NEC.

Innate immunity plays an important role in the initial stages in host defense and appears before the adaptive immune system [10]–[12]. In the mucosal epithelium, it provides rapid protection against microbial invasion [13]. Nucleotide Oligomerisation Domain 2 (NOD2) and Toll-like receptors (TLRs) are important pattern recognition molecules that detect motifs of pathogens and host material released during injury, and activate nuclear factor-κB (NF-κB) pathways leading to the production of antimicrobial immune mediators. Among the TLRs, TLR-2 and TLR-4 are of major importance for several reasons: first, TLR-2 and TLR-4 are overexpressed in human fetal enterocytes [14], [15] whereas their expression is downregulated in 1-month-old rats and they are expressed at very low levels in healthy adult intestinal epithelium; second, they play a major proinflammatory role *in vivo* after ischemia–reperfusion injury and thirdly, soluble TLR-2 which is constitutively expressed in breast milk is a negative regulator of TLR-2. NOD2 can be activated by muropeptides which are components of the bacterial cell wall and although NOD2 mutation are not associated with NEC [16], its expression has not been investigated in this disease. Furthermore, it has been suggested that NOD2 could be a natural inhibitor of TLR-2 [17]. Thus, they could be an important player in the initiation of an exaggerated inflammatory response leading to intestinal injury [18].

In this study, we specifically evaluated intestinal epithelial cells expression of TLR-2, TLR-4, NOD2 andphosphoNF-κB after mucosal injury in a rat NEC model induced by prematurity, systemic hypoxia, and protein rich formula [19].

## Results

### Morphologic small intestinal damage and histological features

**Figure 1:**
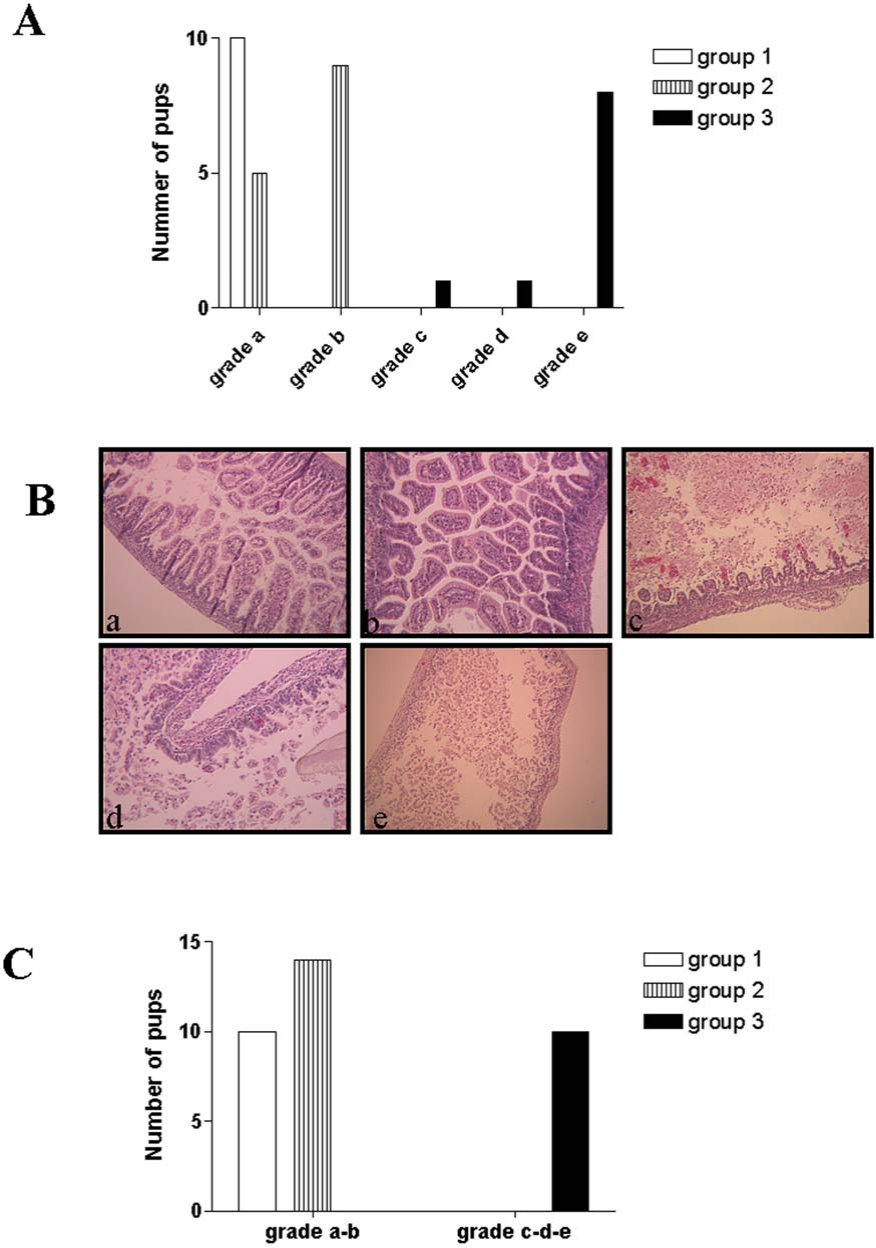
Study of morphological mucosal changes of the small bowel in NEC-induced in rats pups. (a) Histological analysis in the three pups groups (group 1: full term and were mother-fed; group 2: preterm and mother-fed; group 3: preterm, hand-gavaged with a protein rich formula after a hypoxia–reoxygenation procedure). according to the histological classification as showing in (b): intact morphology of the villi (a); sloughing of villi tips (b); mild-villous necrosis (c); loss of villi (d); complete destruction of the mucosa, transmural necrosis (e). (c) Severity of NEC in the different pups groups. Original magnifications: 120×(a,b,c,d), 60×(e).

Morphological changes were analyzed in control neonates rats (group 1: full term and were mother-fed) and in preterm experimental groups (group 2: mother-fed; group 3: hand-gavaged with a protein rich formula after a hypoxia–reoxygenation procedure). With prematurity, general hypoxia and a protein rich cow's milk formula, all of the pups of the group 3 (100%) developed macroscopic changes similar to neonatal NEC, compared with 0% of the control group and 42% of the preterm dam-fed rats. Thus, this model offers a reproducible method for inducing experimental NEC (figure 1a and 1b).

The severest gross lesions (necrosis) were only found in group 3, whereas the bowel remained macroscopically normal in the control group. In group 2, there were hemorrhages in the bowel wall, but no necrosis.

The histological analysis results for the 34 pups are shown in Figure 1, on a scale of (a) to (e). Evaluation of the intestines of the pups in the control group did not reveal any histological signs of mucosal architecture changes. Mild pathological changes—scale (b)—were significantly (p<0.01) observed in the intestines of pups from group 2 versus control. The highest scales (c-d-e) were significantly (p<0.001) observed in group 3 (figure 1c). No perforation was observed. We also analyzed intestines removed from animals that died of NEC, and constantly found hemorrhagic and necrotic villous mucosal lesions (scale (e)). Finally, the macroscopic features correlated with the histological findings in the experimental groups.

### Immunohistochemical analysis of enterocyte proliferation and apoptosis

#### Ki-67 is a relevant marker for assessing cell proliferation and is present throughout the cell cycle and absent in resting cells

**Figure 2:**
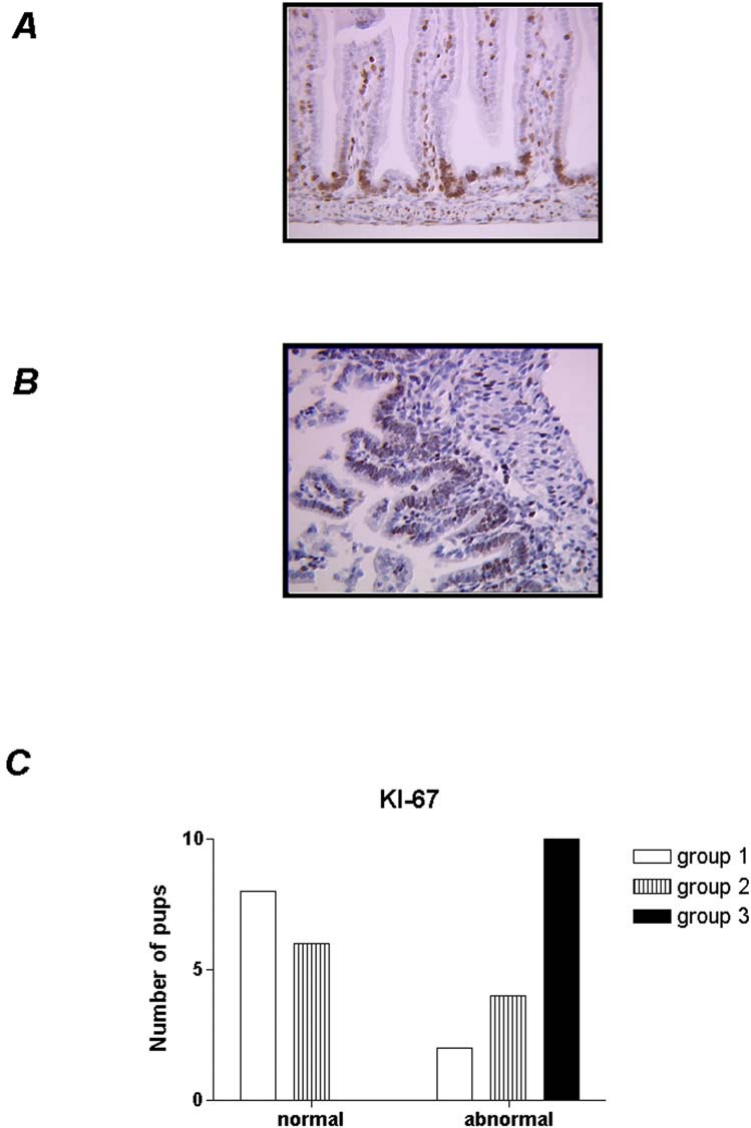
Immunohistochemical features of epithelial cells positive for anti Ki-67 antibody staining in the small bowel mucosa. (a) normal staining is limited to the crypts; (b) abnormal staining extends beyond the crypts, and is continuous, irregular or spreads into the covering villi (*arrowheads*). (c) Histological analysis of KI-67 localisation. Representative images are shown (original magnification 400×)

In all control rats, Ki-67 expression was normally limited to the basis of the crypts (figure 2a). In contrast, as shown in Figure 2b, abnormal Ki-67 expression was observed in both experimental groups. In the preterm pups (group 2), increased numbers of positive nuclei were found, but they were limited to the crypt compartment and not reach statistical significance in comparison to the control group. In contrast, abnormal Ki-67 expression (i.e: positive cells extended beyond the crypts towards the villi) was significantly observed (p<0.01) group (3) in comparison to control group and preterm pups (figure 2c).

**Figure 3:**
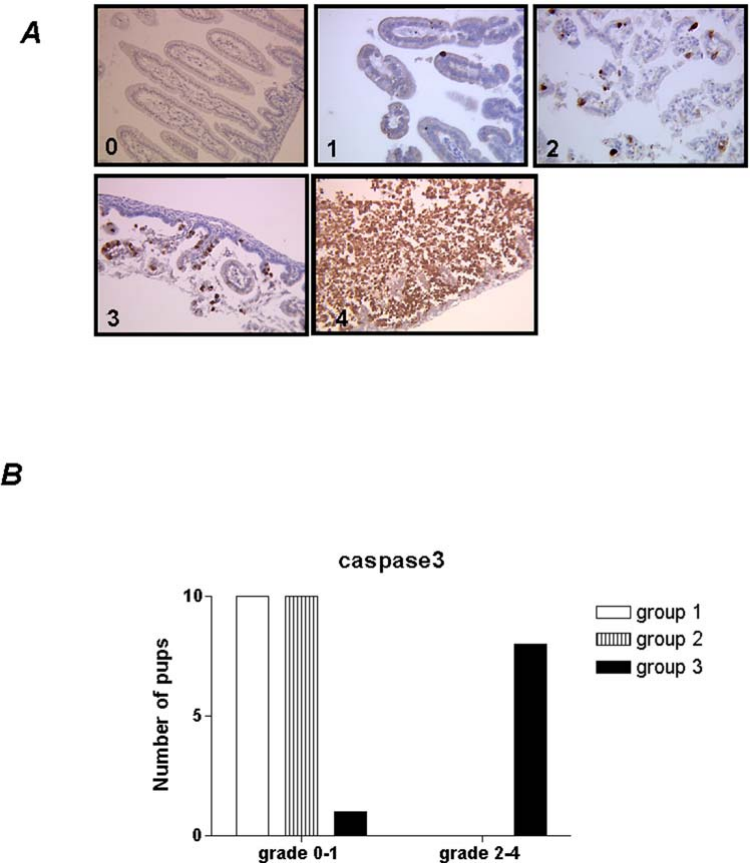
Scales used for the semiquantitation of the small bowel mucosal epithelial cells positive for immunohistochemical staining with an anti caspase-3 antibody. (0): with 0 to 2 cells; (1): with 2 to 5 cells; (2): with 5 to 20 cells; (3): with 20 to 50 cells; (4): more than 50 cells. Representative images are shown (original magnification 400×).

Caspase 3 is involved in apoptotic pathway and the cleaved form of caspase-3 is a specific marker of apoptosis. Therefore, analysis of apoptosis was performed using a antibody against the cleaved form of caspase-3. The grading of the number of caspase-positive epithelial cells is shown in figure 3a. In control and premature groups pups epithelial apoptosis was significantly lower (p<0.01) in comparison with group 3 (figure 3b). In addition, severe histological lesion in the H&E-stained sections were significantly associated with a high grade [(2, 3, 4] of apoptotic cells (figure 3b).

### Immunohistochemical distribution of TLR-2 and pNF-κB p65

**Figure 4:**
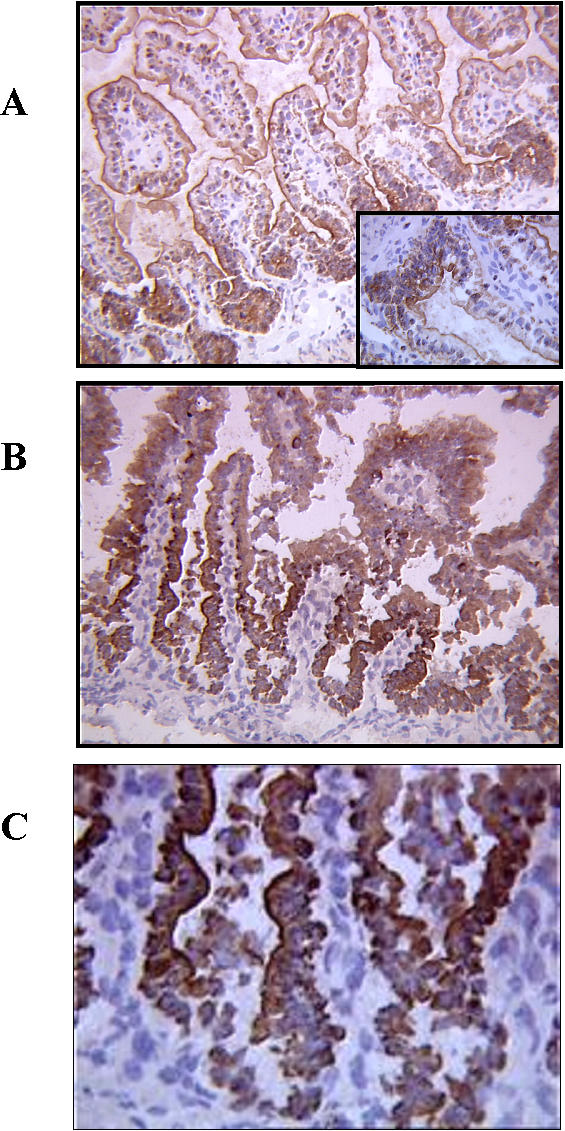
Immunohistochemistry using anti-TLR 2 antibody in ileal samples. (a) In control group, TLR-2 positive cells were located in the crypts, with weak and apical cytoplasmic staining. (b) In group 2 (scored as scale (b) in H&E-stained sections) and (c) in group 3 combining the 3 factors (scored as scale (d) in H&E-stained sections), a strong cytoplasmic TLR-2 staining was observed in the crypts and in the villi (Original magnification 200×, 400×).

As shown in Figure 4, TLR-2 positive cells were constantly observed in normal ileal mucosa. These positive cells were exclusively located in the crypts, with weak, almost apical, cytoplasmic staining (figure 4a) whereas in the groups 2 and 3, strong cytoplasmic TLR-2 staining was observed in the crypts and in the villi (figures 4b and 4c).

**Figure 5:**
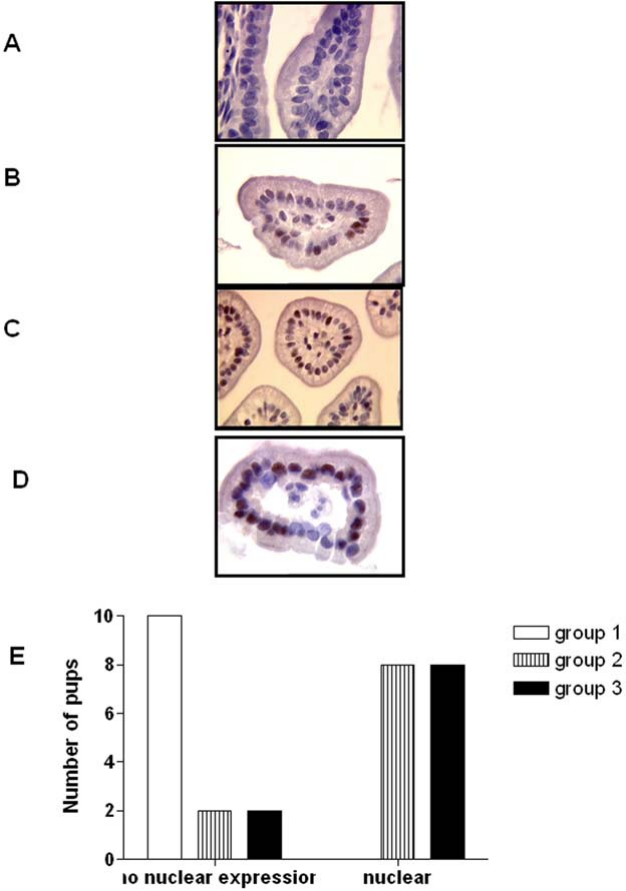
Immunohistochemistry using anti anti-pNF-κB antibody in ileal samples. In group 1, there is no nuclear staining in an intestinal villous (a and e) whereas in group 2 (scored as scale (b) in H&E-stained sections), some positive cells are observed (b). These positive cells are much more numerous in the group 3 (scored as scale (c) in H&E-stained section) (c and d). (Original magnification ×400 and 800 respectively).

As shown in figure 5a, nuclear pNF-κB p65 expression was never detectable in the control group whereas nuclear staining were significantly detected in experimental groups 2 and 3 in 50% and 80% respectively (p<0.001). Furthermore, mean of positive cells per 100 epithelial cells was 12.6% in group 2 and 25% in the group 3 (p<0.05).

Thus, in the epithelial cells from pups suffering from NEC there is up-regulation of expression of molecules involved in innate immunity when compared with pups from groups 1 and 2.

### Real-time RT-PCR of TLR-2, TLR-4 and NOD2

**Figure 6:**
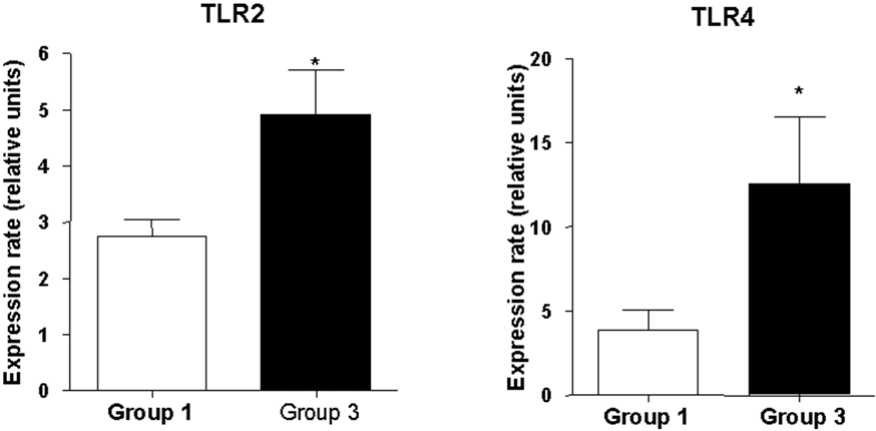
mRNA expression levels of TLR-2 and TLR-4 from distal jejunum were analysed by real-Time-PCR under basal and NEC conditions. (a) TLR-2 and (b) TLR-4 mRNA expression levels from distal jejunum. Data represent the means±SEM of 10 mice per group. *P<0.05 and **P<0.01, significantly different from group 1.

We investigated at mRNA levels TLR-2, TLR-4 and NOD2 expression using real-time RT-PCR in the distal jejunum. In agreement with immunohistochemical data, we show that mRNA levels of TLR-2 and TLR-4 were increased by 72% and 140%, respectively in group 3 (figure 6). In contrast, we failed to reveal any difference in NOD2 mRNA expression between groups 1 and 3 (data not shown).

## Discussion

We successfully produced a neonatal model of NEC in rats that is close to the human pathogenesis hypothesis. Therefore, we use a combination of the three main factors likely to induce clinical and pathological NEC damage [20]–[22]: prematurity, a rich cow's milk-based milk substitute and systemic hypoxia. The rat model of NEC is usually obtained using ischemia induced by mesenteric artery ligature. However, these previous procedures have some limitations. First, they do not exactly reproduce the features usually observed in human NEC, and second, they are not able to define precisely the mechanisms of bowel injury. Our model is adapted from the one developed by Caplan [4], [5], [23], [24] using an oral feeding procedure with hand-gavages [25]. We provide slight modifications to this rat model by using a particularly protein rich formula adapted to rodents to initiate intestinal barrier mucosa injury. Considering that intestinal hypoxia/ischemia-reperfusion in the perinatal period appears to be one of the contributing factors to development of NEC, experimental NEC was induced by exposure to hypoxia induced by 100% CO2 instead of nitrogen and reoxygenation in 100% oxygen followed by a cold stress, both involving in alteration of mucosal barrier and permeability.

Thus, this model led to macroscopic (distension, discoloration, hemorrhage, or wall necrosis) and microscopic features close to human NEC. Interestingly, the frequency and severity of NEC was higher in group 3, in which all of the factors were combined. We also observe that in cases of prematurity and feeding with rat milk the severity of the NEC was decreased but not totally prevented, as was previously demonstrated in humans [6], [26]. Thus, prematurity alone causes little or no histological lesions. These data confirm that NEC may indeed result from a combination of several risk factors [27] that act synergistically. We assume that prematurity could play an initiatory role in NEC, with immaturity of mucosal barrier. Innate immune activation is initiated by bacterial colonization at the mucosal surface. Insofar as NEC occurs in the same circumstances as those which lead to innate immunity activation (epithelial cell layer injury), it was interesting to evaluate the expression of molecules involved in innate immunity in NEC.

The aim of our model was to allow the harvest of intestinal tissue with NEC lesions to extend our study to immunohistochemical analysis of the role of innate immunity in NEC. This model induces NEC with histological abnormalities, the severity of which can be modulated. Epithelial intestinal cell proliferation, apoptosis, and the expression of molecules involved in innate immunity can be evaluated using different scales in the group 3. We showed that intestinal epithelial cell proliferation, as assessed by Ki-67 expression, and apoptosis, assessed by caspase-3 activity, were altered and correlated to the highest scales in the H&E-stained sections. Interestingly, these immunohistochemical alterations appear before the clinical signs or gross changes of NEC are evident. Furthermore, they are present even in apparently intact villi, whereas only scattered apoptotic cells were observed in the control group. These results are in accordance with previous studies in a comparable NEC model [20] and in ischemia–reperfusion and bacterial invasion NEC models [28]. NF-κB plays a pivot role in innate immunity, participating in the inflammatory cascade. Its expression was evaluated using an anti-pNF-κB antibody that recognizes the activated nuclear form of NF-κB. As for apoptosis, we observed an increase in positive NF-κB epithelial cells. The number of positive cells was correlated with the severity indicated by the scoring of the H&E sections. Interestingly, TLR-2 and pNF-κB expression was strongly upregulated in the IECs from the group 3 pups with NEC. However, the most striking feature was profound perturbation of the immunostaining pattern, with overexpression of TLR-2 in the villi and the crypts and in the whole cytoplasm. TLR2 and pNF-κB expression were marked in the group 3 animals, suggesting that this immune response is exacerbated in NEC. Recently, De Plaen et al found that intestinal NF-KappaB is strongly activated at birth, is down-regulated within a day and remains activated in a neonatal model of NEC [29]. We also analyse TLR4 in neonatal NEC because TLR4 is the receptor for LPS, the most common bacterial-derived product that has been implicated in NEC. Increase of TLR2 and TLR4 mRNA expression has been reported in fetal human intestine [15], but normal adult human intestine exhibits little to no TLR2 and TLR4 expression. It has been shown that LPS responsiveness rapidly declines after birth in primary murine enterocytes [30] As for TLR-2, TLR-4 expression was also increased in NEC using real time RT-PCR. In accordance with our results, it has been shown intestinal that TLR4 mRNA gradually decreases in mother-fed but increases in formula feeding and cold asphyxia stress in a experimental mice NEC model, where TLR4 deficiency appears to be protective [31]. In contrast, Nod2 expression is unchanged in intestinal samples. In humans, two genetic studies tested the association between NOD2 and risk of NEC showing that genetic polymorphisms of Nod2 are not associated with NEC or prematurity [16], [32].

These results suggest that in the preterm intestinal mucosa, upregulation of the innate responses occurs early before histological lesions arise. The immature fetal small intestine has an excessive response to mucosal injury compared with the small intestines of full-term pups. Prematurity may thus render the neonatal digestive tract more susceptible to mucosal barrier failure and lead to NEC if associated with other risk factors. The IEC phenotype obtained in this rat model of NEC is very close to those observed in fetal IEC, thus suggesting that NEC could result in abnormal maintenance of a fetal phenotype in IEC. Host-intraluminal bacteria interactions and bacterial-derived products activate TLR on intestinal epithelial cells that are abnormally up-regulated following neonatal stress, resulting in downstream inflammatory gene expression and NEC and bowel injury.

## Materials and Methods

### Animal model of NEC

All procedures were performed in accordance with the institutional animal care and use committee (No 87-848, 19 October 1987). Thirty-four newborn rats were obtained from timed pregnant Wistar rats (Charles River Laboratories, l'Arbresles, France). The pups were divided into three groups:

-group 1 (control group, n = 10): the neonatal rats were obtained after spontaneous vaginally delivery at full term and were mother-fed;

-groups 2 and 3 (experimental groups): the pups were obtained by preterm delivery by caesarean section at day 21 of pregnancy. Group 2 (n = 14) were then breast-fed, and group 3 (n = 10) were stressed at 1 hour of life with asphyxia induced by breathing 100% CO_2_ for 5 minutes with simultaneous exposure to cold (1°C) followed by reoxygenation with 100% O_2_ for 5 minutes. In addition, 2 hours after this hypoxia–reoxygenation procedure, the newborn rats were hand-fed via an oral feeding tube (20G) with 0.15 ml formula every 4 hours during the first 48 hours of life. The rat-milk substitute was based on TVM® formula (TVM Laboratories, Lempdes, France) supplemented with 12 g/100 ml of protein (Resource Protein Instant®, Novartis Nutriton, Revel, France) to give a protein rich formula.

### Tissue harvest and NEC evaluation

Following incision of the abdomen, the small intestine was evaluated visually for typical gross signs of NEC such as intestinal distension, intestinal wall hemorrhages, or necrosis. The entire small intestine was harvested and 20-mm lengths of the distal jejunum and distal ileum were taken. Half of each sample was immediately snap frozen in nitrogen for the immunohistochemical and RT-PCR studies, whereas the other half was formalin-fixed, paraffin-embedded, microtome-sectioned at 5 μm, and stained with hematoxylin and eosin (H&E) for histological evaluation.

The morphological changes in the mucosa seen in the H&E-stained sections were scored and graded on the scale of (a) to (e) as follows: (a) intact villi, (b) sloughing of villi tips, (c) mild necrosis of villi, (d) loss of villi, and (e) complete destruction of the mucosa or transmural necrosis . This scoring was adapted from the system described by Jilling *et al.* [20]. The score was determined based on the highest score observed in a specimen. Only the specimens that were categorized as score (c, d and e) were regarded as severe enterocolitis.

To explore the spectrum of mucosal damage, we performed immunohistochemistry on paraffin sections and corresponding frozen sections of jejunal samples.

### Immunohistochemistry for Ki-67, caspase-3, pNF-κB, and TLR-2 expression

Slides were prepared such that the H&E-stained and the immunohistochemistry slides were from the same block. After being deparaffinized, sections were rehydrated in serial dilutions of ethanol and water; antigen unmasking was achieved using hot shock (for the antiphosphoNF-κB p65 antibody), followed by blocking of endogenous peroxidase using 3% hydrogen peroxide for 10 minutes. Sections were then washed twice in Tris HCl, pH 7.6 and briefly in buffer containing 1% polymerized bovine albumin.

The following antibodies were used for the identification of Ki-67 and phosphoNF-κB–positive epithelial cells: rabbit polyclonal anti-Ki-67 (Novacastra), rabbit antibody anti-cleaved-caspase-3 (IGG polyclonal; Cell Signaling Technology), rabbit antibody anti-phosphorylated-NF-κB (pNF-κB p65, Cell Signalling Technology) that recognizes the activated nuclear form.

For TLR-2, sections of frozen tissue were cut, fixed in 100% acetone and stored at −20°C. A goat anti-mouse TLR-2 polyclonal antibody (Santa Cruz Biotechnology) was used for the identification of TLR-2-positive epithelial cells. Sections were washed twice in Tris HCl, pH 7.6 and briefly in buffer containing 1% polymerized bovine albumin.

The sections were incubated for 1 hour at room temperature with the primary antibodies diluted 1:600 for Ki-67), 1∶100 for caspase-3, 1∶25 (for pNF-κB p65, and 1∶100 for TLR-2. Ki-67, caspase-3, pNF-κB p65, and TLR-2 were detected using the Elite Kit ABC for rabbit or goat from Vectastain, in which the secondary antibody is biotinylated and the label is peroxidase-conjugated streptavidin. Control sections were treated with the same procedure except they were incubated without the specific primary antibodies.

### Semi-quantitative analysis of positive enterocytes in the immunochemistry study

The distribution of Ki-67 expression was considered as normal when the staining remained confined to the crypts and abnormal if the staining was beyond the crypts and spread to the covering villous (Figure 2).

The distribution of caspase-3 expression was scored on a four level scale by counting 10 microscopic fields at ×40 magnification: scale (0), 0 to 2 positive cells; scale (1), 2 to 5 positive cells; scale (2), 5 to 20 positive cells, scale (3), 20 to 50 positive cells, and scale (4), more than 50 positive cells.

The distribution of TLR-2-positive epithelial cells was evaluated in the epithelial cell layer (limited in the crypts, or spread to the surface of the villi) and into the subcellular compartment.

The distribution of pNF-κB p65 positive epithelial cells was evaluated by counting the nuclear staining per 100 epithelial cells at ×40 magnification

### Real time reverse transcription-polymerase chain reaction (RT-PCR)

After extraction from distal jejunum by the NucleoSpin® RNA II Kit (Macherey-Nagel, Hoerdt, France), total RNA was converted to cDNA using random hexonucleotides and then used for PCR. We conducted PCR with QuantiTect SYBR Green PCR Kit (Applied, Courtaboeuf, France) using sense and antisense primers specific for: G3PDH, 5′-AGAGAGAGGCCCTCAGTTGCT-3′ and 5′-TGGAATTGTGAGGGAGATGCT-3′; Toll like receptor 2 (TLR2), 5′-GTACGCAGTGAGTGGTGCAAGT-3′ and 5′-GGCCGCGTCATTGTTCTC-3′; Toll like receptor 4 (TLR4), 5′-AATCCCTGCATAGAGGTACTTCCTAAT-3′ and 5′-CTCAGATCTAGGTTCTTGGTTGAATAAG-3′, NOD2 5′-TTCTGCCTTACGAGGGTTACTCTCT-3′ and 5′-ATGGTCCTCAGCTTAGCAGTGAAC-3′. After amplification, we determined the threshold cycle (Ct) to obtain expression values of 2^−ΔΔct^, as previously described [32].

### Statistical Analyses

To determine the incidence of the prematurity and the combination of prematurity, protein rich formula and a hypoxia–reoxygenation procedure on the morphological changes of jejunal mucosa and, Ki-67, pNF-κB p65 and caspase 3 expression, a contingency table and Fisher's exact test were used to analyse the differences. However the incidence of the combination of prematurity, protein rich formula and a hypoxia–reoxygenation procedure on the TLR-2 and TLR-4 mRNA expression, single comparisons were performed by unpaired Student's t-test. Statistical analysis were performed using GraphPad Prism 4.00 (GraphPad Software, San Diego, CA, USA) software package for PC. Differences were considered significant at P<0.05 and All P values were two sided.
